# A Model of Metabolic Syndrome and Related Diseases with Intestinal Endotoxemia in Rats Fed a High Fat and High Sucrose Diet

**DOI:** 10.1371/journal.pone.0115148

**Published:** 2014-12-11

**Authors:** Xin Zhou, Dewu Han, Ruiling Xu, Suhong Li, Huiwen Wu, Chongxiao Qu, Feng Wang, Xiangyu Wang, Yuanchang Zhao

**Affiliations:** 1 Department of Pathophysiology, Basic Medical Science, Shanxi Medical University, Taiyuan, 030001, Shanxi, China; 2 Department of Pathology, Shanxi Tumor Hospital, Taiyuan, 030013, Shanxi, China; 3 Science & technology center of Fenyang College, Shanxi Medical University, Fenyang 032200, Shanxi, China; 4 Department of Pathology, Shanxi Provincial People's Hospital, Taiyuan, 030012, Shanxi, China; 5 Department of Oral Medicine, Shanxi Medical University, Taiyuan, 030001, Shanxi, China; University of Catania, Italy

## Abstract

**Aim:**

We sought develop and characterize a diet-induced model of metabolic syndrome and its related diseases.

**Methods:**

The experimental animals (Spague-Dawley rats) were randomly divided into two groups, and each group was fed a different feed for 48 weeks as follows: 1) standard control diet (SC), and 2) a high sucrose and high fat diet (HSHF). The blood, small intestine, liver, pancreas, and adipose tissues were sampled for analysis and characterization.

**Results:**

Typical metabolic syndrome (MS), non-alcoholic fatty liver disease (NAFLD), and type II diabetes (T2DM) were common in the HSHF group after a 48 week feeding period. The rats fed HSHF exhibited signs of obesity, dyslipidemia, hyperglycaemia, glucose intolerance, and insulin resistance (IR). At the same time, these animals had significantly increased levels of circulating LPS, TNFα, and IL-6 and increased ALP in their intestinal tissue homogenates. These animals also showed a significant reduction in the expression of occluding protein. The HSHF rats showed fatty degeneration, inflammation, fibrosis, cirrhosis, and lipid accumulation when their liver pathologies were examined. The HSHF rats also displayed increased islet diameters from 12 to 24 weeks, while reduced islet diameters occurred from 36 to 48 weeks with inflammatory cell infiltration and islet fat deposition. The morphometry of adipocytes in HSHF rats showed hypertrophy and inflammatory cell infiltration. HSHF CD68 analysis showed macrophage infiltration and significant increases in fat and pancreas size. HSHF Tunel analysis showed significant increases in liver and pancreas cell apoptosis.

**Conclusions:**

This work demonstrated the following: 1) a characteristic rat model of metabolic syndrome (MS) can be induced by a high sucrose and high fat diet, 2) this model can be used to research metabolic syndrome and its related diseases, such as NAFLD and T2DM, and 3) intestinal endotoxemia (IETM) may play an important role in the pathogenesis of MS and related diseases, such as NAFLD and T2DM.

## Introduction

The intestinal lumen presents the largest surface area within the human body for bacteria to colonize and produce potential toxins; the lipopolysaccharide (LPS)-containing membranes of Gram-negative bacteria represent an innate toxin, referred to as endotoxin [1]. Under normal physiological conditions, endotoxin is continuously released into the intestinal lumen, but it does not exhibit any pathogenic effects [1]. Typically, a portion of the released endotoxin is absorbed into the portal circulation and delivered to the liver, where it is quickly cleared by intrahepatic Kupffer cells [1]. However, when the body suffers severe trauma, systemic infections, intestinal ischemia, and/or liver disease, a large amount of the endotoxin from the intestine will translocate into the systemic blood causing intestinal endotoxemia (IETM) [2], which is correlated with the overgrowth of Gram-negative organisms within the intestine, increased intestinal permeability, and impaired Kupffer cell phagocytic functions [2].

In the past 20 years, Han's laboratory has been one of the leading groups studying IETM. Han et al. have conducted a series of animal and human studies documenting that IETM is often found concomitantly with various types of liver diseases, including viral hepatitis, alcoholic liver disease, chemical and drug-induced liver injury, and hepatic failure [3], [4]. Recent data indicate that non-alcoholic fatty liver disease (NAFLD) and type II diabetes mellitus (T2DM) are also associated with increased plasma endotoxin levels [5]–[8]. Both of these diseases represent serious and often fatal health conditions that are prevalent throughout the world.

It has been well-established that NAFLD and T2DM are metabolic diseases [9], [10]. Consumption of foods containing high fat and high sucrose, which are frequently associated with a “western diet,” account for the largest incidence of metabolic syndrome and its related diseases [11], [12]. “Over-nutrition” can alter the gut microbiota by changing the available nutrient sources, while also increasing the intestinal permeability [13]. Therefore, it stands to reason that regular consumption of a “western diet” could lead to intestinal endotoxemia. Some researchers have suggested that intestinal endotoxemia may be involved in the development of a chronic low-grade inflammatory state in the host that contributes to the development of metabolic syndrome, which can be associated with chronic diseases, such as NAFLD and T2DM [6], [7] [14], [15]. However, it is currently unknown as to how changes in endotoxin levels induce metabolic syndrome and its related diseases; most studies on the topic involve the administration of copious amounts of purified bacterial LPS in a rodent model of IETM that does not accurately model the clinical conditions common in patients with metabolic diseases [15].

Our previous work has utilized a rodent model where rats will develop insulin resistance and nonalcoholic steatohepatitis by the 9^th^ week following subcutaneous injection of small doses of LPS [16]. Thus, we hypothesized that IETM may play a central role in the development of metabolic syndrome by causing chronic low-grade inflammation and associated metabolic disorders. Therefore, the goal this work was to establish a model of metabolic syndrome (MS) and related NAFLD and T2DM where IETM occurs spontaneously, so that we could research the role of intestinal endotoxemia in the development of non-alcoholic fatty liver and type II diabetes. We ultimately found that rats fed a high fat and high sucrose diet for 48 weeks gradually developed metabolic syndrome and its associated diseases, which appears to be related to the occurrence of IETM.

## Materials and Methods

### Animals and diets

Sixty-four male Spraque-Dawley rats weighing 200–250 g were obtained from the Animal Center of Shanxi Medical University. All animals received humane care during the experiment under a protocol that was approved by the Committee on Animal Research and Ethics of Shanxi Medical University (Permit Number: SCXK (JIN) 2009–0001). All surgeries were performed under sodium pentobarbital anesthesia, and all efforts were made to minimize suffering. The experimental animals were randomly divided into two groups (*n* = 32 for each group); each group was fed a different chow for 48 weeks. One group was feed a high sucrose and high fat diet (HSHF; 65% of calories from carbohydrates, 25% from fat, and 10% from protein), while the other was fed standard chow (SC; 61% of calories from carbohydrates, 7% from fat, and 15% from protein). Fresh chow was supplied daily before dark, and animals were allowed to drink water freely. The caloric content of food intake was measured based on standard chow with a calorie content of 13.9 kJ/g and HSHF chow with a calorie content of 68.4 kJ/g. The body mass of each animal was weighed monthly throughout the feeding period.

The Rats fasted for 12 hours and were then sacrificed at 12, 24, 36, and 48 weeks. Then the blood, small intestine, liver, pancreas, and fat tissues (epididymal and perirenal fat pads) were sampled following sacrifice.

### Measurements of serum ET and ALP activity in small intestine tissue and Biochemical analysis

The level of endotoxin in plasma was determined using a Limulus kit (Clinical Sciences Inc, Xiamen China) according to the manufacturer's instructions. The levels of ALP (Alkaline phosphatase kits, Nanjing Jiancheng Bioengineering Institue, Nanjing China) in 1% of each intestinal homogenate were measured according to the kit instructions.

The contents of Tch (total cholesterol kit, Nanjing Jiancheng Bioengineering Institue, Nanjing China) and TG (total triglycerides kits, Nanjing Jiancheng Bioengineering Institue,Nanjing China) were measured by the colorimetric enzymatic method. The contents of HDL-ch (high density lipoprotein-cholesterol kits, Nanjing Jiancheng Bioengineering Institue, Nanjing China) were assayed using the phosphotungstic acid magnesium chloride precipitation method, according to the manufacturer's instructions. The contents of LDL-ch (low density lipoprotein-cholesterol kits, Nanjing Jiancheng Bioengineering Institue, Nanjing China) were assayed using the enzyme method. The enzyme alanine transaminase levels (ALT, alanine transferase kits, Nanjing Jiancheng Bioengineering Institute, Nanjing China) were measured in the plasma of the animals according to the manufacturer's instructions.

### Intraperitoneal glucose tolerance test (IPGTT)

Fasting blood glucose was measured (12 h fast, blood taken from the tail vein) using a glucose meter (Roche Diagnostics GmbH, Mannheim, Germany). Glucose (2 g/kg rat) was then injected intraperitoneally, and blood glucose was determined again at 30, 60, 90, and 120 min post-injection.

### Radioimmunoassay for plasma insulin and HOMA-IR, and HOMA-β

Plasma insulin concentrations was determined using an insulin RIA kit (Purevalley biotech, beijing china). The HOMA-IR (homeostasis model assessment of insulin resistance) index was calculated as [fasting serum glucose × fasting serum insulin/22.5] to assess insulin resistance. The HOMA-β (homeostasis model assessment of β-cell function) index was calculated as [20× fasting serum insulin]/[fasting serum glucose- 3.5] to assess β-cell function.

### Radioimmunoassay for TNFα and IL-6 in plasma and TNFα in pancreas and fat tissue

Tissue (500 mg) was removed from the pancreas and epididymal fat pads, and 1 ml saline was added to make homogenates in an ice bath. Samples were centrifuged for 15 minutes (12000 rpm) and kept at -20°C degrees to save the supernatants.

Levels of tumor necrosis factor (TNFα) and interleukin 6 (IL-6) concentrations were determined using an TNFα RIA kit and IL-6 RIA kit, respectively (Purevalley biotech, Beijing China).

### Histology and Lee's index, liver index, and fat index

Samples from intestinal tissue, epididymal fat pads, liver, and pancreas were fixed in 10% buffered formalin, embedded in Paraffin, cut into 4 µm thick sections, and stained with hematoxylin and eosin (HE Staining Kit, Junruishengwu Technnology Coporation, Shanghai China). Pathological changes occurring in the intestinal tissue, fat pads, liver, and pancreas were examined under light microscopy (Olympus BX51 microscope, 100X magnification). After examining a consecutive series of sections, we chose to record the islet morphology index of one slice in every six to seven to avoid selecting a slice in a similar region [3]. Using the Olympus BX51 system to collect photos, we measured islet maximum diameter in these images using Image-Pro Plus version 5.0 (Media Cybernetics Inc., USA) with a sample size of at least 200 islets per group. To measure the cross-sectional area of adipocytes, at least 50 adipocytes per animal were evaluated with the software Image-pro Plus version 5.0. Frozen sections of liver and pancreas were sliced and stained with Sutan IV (Sudan IV fat staining kit, Junruishengwu Technnology Coporation, Shanghai China) and examined at 100X magnification.

The body weight and the length of the rats were recorded and used to calculate Lee's index (body weight (g)^1/3^×1000/body length (cm)). Under sterile conditions, we removed and weighed the liver and adipose tissue (epididymal and perirenal) and calculated the ratio of the fat (epididymal and perirenal) and liver weight (g) to body weight (kg).

### Lipid Content and Hydroxyproline (HYP) Content of the Liver and pancreas

Hepatic lipids were extracted as previously described [17]. The lipid extracts were resuspended in methanol and used for the measurement of free fatty acid levels (FFA Kit, Nanjing Jiancheng Bioengineering Institue, Nanjing China). The levels of HYP (Hydroxyproline kits, Nanjing Jiancheng Bioengineering Institue, Nanjing China) in 1% of each liver and pancreas homogenate were measured according to the kit instructions.

### Immunohistochemistry

Paraffin-embedded pancreas and epididymal fat pad sections were stained with CD68 protein adducts using a polyclonal antibody (1∶100 dilution; Zhong Shan-Golden Bridge Biological Technology Co. Beijing, China) to observe quantitative expression levels of CD68.

The specimens were analyzed using a computerized image analysis system (IPP6.0 software, Media Cybernetics Inc., USA). At least 5 horizons per animal were calculated. To determine means, data were displayed as the number of CD68-positive cells/number of total adipose cells (x 400).

### Terminal Deoxynucleotidyl TransferaseMediated dUTP Nicked End Labeling (TUNEL) Assay

TUNEL was used for in situ detection of apoptosis in the liver and pancreas. Tissues were paraffin dewaxed to water, washed in phosphate buffered saline (PBS), pH 7.4 twice for 5 min each time, then incubated in 3% hydrogen peroxide for 20 minutes. Samples were then PBS washed twice more and dried. Reaction solution (TUNEL kits, Roche Diagnostics GmbH, Germany) was added to each sample (50 µl), and the reactions were performed at 37°C for 60 min in the dark. After incubation, samples were again washed twice in PBS and 50 µl Converter-POD (TUNEL kits, Roche Diagnostics GmbH, Germany) was added to the sample and incubated at 37°C for 30 min. Samples were washed again in PBS and a DAB Horseradish Peroxidase Color Development Kit (Beyotime Institute of Biotechnology, Shanghai China) was used to stain the samples. Hematoxylin was used to stain the nuclei. Samples then underwent conventional dehydration and were transparently mounted with glycerol. Colored nuclei were read as positive cells. The specimens were analyzed using a computerized image analysis system (IPP6.0 software, Media Cybernetics Inc, USA), and the data were represented as the number of TUNEL-positive cells per field (at 400X).

### Western blot analysis

Total occludin was assessed by Western blot. Aliquots of frozen intestinal tissue homogenates were further extracted in phosphate-buffered saline containing of 1% NP-40, 0.5% sodium deoxycholate, 0.1% SDS, 0.1 mM EDTA, 50 mM NaF and 2 mM Na_3_VPO_4_ (NaF and Na_3_VPO_4_ only for phosphorylation protein; chemicals come from Nanjingjiancheng Biotechnology, Nanjing China, and lysed by 30 min incubation on ice. The lysate was centrifuged at 15,000 rpm for 10 min. Forty µg of protein was loaded in each lane and separated on a 7.5% sodium dodecyl sulfate polyacrylamide gel (SDS-PAGE) and then transferred to a polyvinylidene difluoride (PVDF) membrane. Blots were blocked for 3 h at room temperature with 5% (w/v) non-fat dried milk. After washing 3 times with TBST (Tris50 mmol/L Nacl100 Mmmol/L PH 7.40), the membrane was incubated at 4°C overnight with specific antibodies. A rabbit polyclonal antibody against occludin (1∶1000 dilution; Cell Signaling Technology Inc. Danvers, USA) was used in the study. The immunoblots were then incubated with the corresponding peroxidase-conjugated goat anti-rabbit secondary antibody (1∶2000 dilution; Zhong Shan-Golden Bridge Biological Technology). The bands were detected with enhanced chemiluminescence. The intensities of the protein bands were analyzed by Quantity One software (Bio-Rad Laboratories, Inc. Hercules, USA).

### Statistics

All values are displayed as means ± SE, and statistical analyses were performed with the SPSS14.0 system (Statistical Product and Service Solutions, USA). Comparisons between the normal group and each model group were made using nonparametric test. Comparisons between the model groups were analyzed by one way analysis of variance (ANOVA). Differences in morphological measures of pancreatic islets and fat cell were analyzed with a non-parametric Kruskal-Wallis test and a p<0.05 was considered statistically significant.

## Results

### Animal characteristics

**Figure 1:**
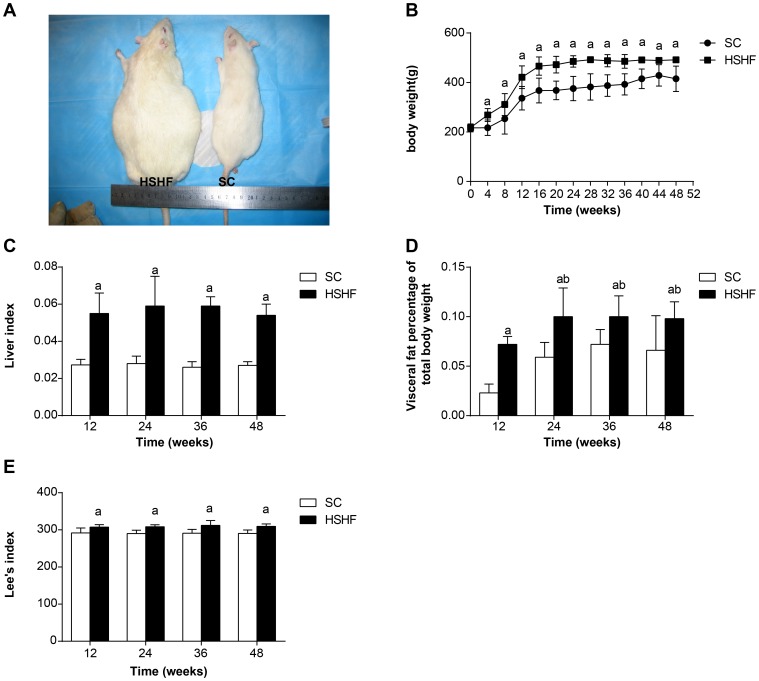
The changes of weight, Lee's index, liver index and fat percentages in different groups per month. A. Comparison of rats. B. Comparison of body weight. C. Comparison of liver index. D. Comparison of fat percentages. E. Comparison of Lee's index. SC: standard chow group; HSHF: high sugar and high fat diet group; ^a^ p<0.05 vs normal control; ^b^ p<0.05 vs 12^th^ week group; ^c^ p<0.05 vs 24^th^ week group; ^b^ p<0.05 vs 36^th^ week group.

The average body weight of HSHF rats was higher than SC rats (Fig. 1A). Significant body weight differences were noted as early as the 4^th^ week (Fig. 1B). By the 48^th^ week, the body weight of HSHF rats was more than 26% greater than SC rats (p = 0.002). Likewise, the Lee's index (Fig. 1C), the fat:body weight ratio (Fig. 1D) and liver:body weight ratio (Fig. 1E) in HSHF rats were all significantly higher than in SC rats (+97%, p = 0.005; +58%,p = 0.039; +90%, P = 0.002).

### Blood biochemistry

Significant differences in plasma levels of triglycerides (TG), cholesterol, high density lipids-cholesterol (HDL-C), and low density lipids-cholesterol (LDL-C) were noted as early as the 12^th^ week, and this difference continued until the end of the experiment (48 weeks). At the 48^th^ week, plasma levels of TG (p = 0.001), cholesterol (p = 0.001), and LDL-C were higher, while the plasma levels of HDL-C (p<0.001) were lower in HFHS rats compared to the SC group (Table 1). The concentrations of ALT were also increased in the HFHS group (p = 0.001, Table 1).

**Table 1:** Blood biochemistry in different group (x±s. n = 8).

Indexes	Groups	12^th^ week	24^th^ week	36^th^ week	48^th^ week
Glucose,mmol/L	SC	5.62±0.57	5.57±0.57	5.74±0.46	5.91±0.33
	HSHF	12.61±0.81^a^	11.80±1.21^ab^	13.85±0.57^abc^	14.57±0.84^abcd^
Insulin, mU/L	SC	19.12±3.62	19.83±2.81	19.24±3.84	20.01±3.23
	HSHF	54.09±15.20^a^	91.28±32.68^ab^	55.20±10.18^ac^	36.15±9.60^abcd^
HOMA-IR index	SC	4.81±1.23	4.95±1.08	4.87±0.79	5.26±0.95
	HSHF	30.25±8.47^a^	47.67±16.43^ab^	28.60±14.75^ac^	23.52±6.93^ac^
HOMA-βindex	SC	190.22±46.90	200.43±38.88	181.80±64.72	169.0±32.57
	HSHF	120.03±37.05^a^	224.75±91.83^b^	87.93±44.37^ac^	65.28±16.24^abc^
Cholesterone,mmol/L	SC	1.82±0.23	1.76±0.15	1.76±0.22	1.87±0.43
	HSHF	3.83±0.75^a^	3.83±1.16 a	3.82±1.02^a^	3.77±0.50^a^
Triglycerides, mmol/L	SC	0.088±0.015	0.083±0.02	0.075±0.012	0.083±0.020
	HSHF	0.61±0.13^a^	0.58±0.07^a^	0.583±0.11^a^	0.597±0.052^a^
LDL-c, mmol/L	SC	0.59±0.13	0.60±0.04	0.60±0.11	0.64±0.08
	HSHF	3.27±0.33^a^	3.31±0.49^a^	3.36±0.30^a^	3.31±0.17^a^
HDL-c, mmol/L	SC	1.76±0.39	1.56±0.30	1.62±0.43	1.61±0.29
	HSHF	0.54±0.04^a^	0.5±0.07^a^	0.48±0.14^a^	0.47±0.11^a^
ALT,U/L	SC	29.19±2.62	27.19±4.25	25.89±2.26	25.89±4.41
	HSHF	259.79±76.11^a^	308.66±24.38^a^	305.61±36.87^a^	310.32±29.40^a^

a p<0.05 vs normal control; ^b^ p<0.05 vs 12^th^ week group; ^c^ p<0.05 vs 24^th^ week group; ^d^ p<0.05 vs 36^th^ week group.

### Homeostasis model assessment of insulin resistance and β-cell function

Significant differences in fasting plasma glucose and insulin were recognized as early as the 12^th^ week and continued for 48 weeks. At the 48^th^ week, plasma levels of glucose (p<0.001) and insulin (p<0.001) were higher in HSHF rats compared to SC rats (Table 1). From the 12^th^ to 48^th^ week, HSHF rats showed increased HOMA-IR (p<0.001), while HOMA-β progressively decreased after a compensatory increase at the 24^th^ week in HSHF rats; HSHF rats also displayed reduced β-cell numbers and decreased β-cell functions (Table 1).

### Intraperitoneal glucose tolerance test (IPGTT)

**Figure 2:**
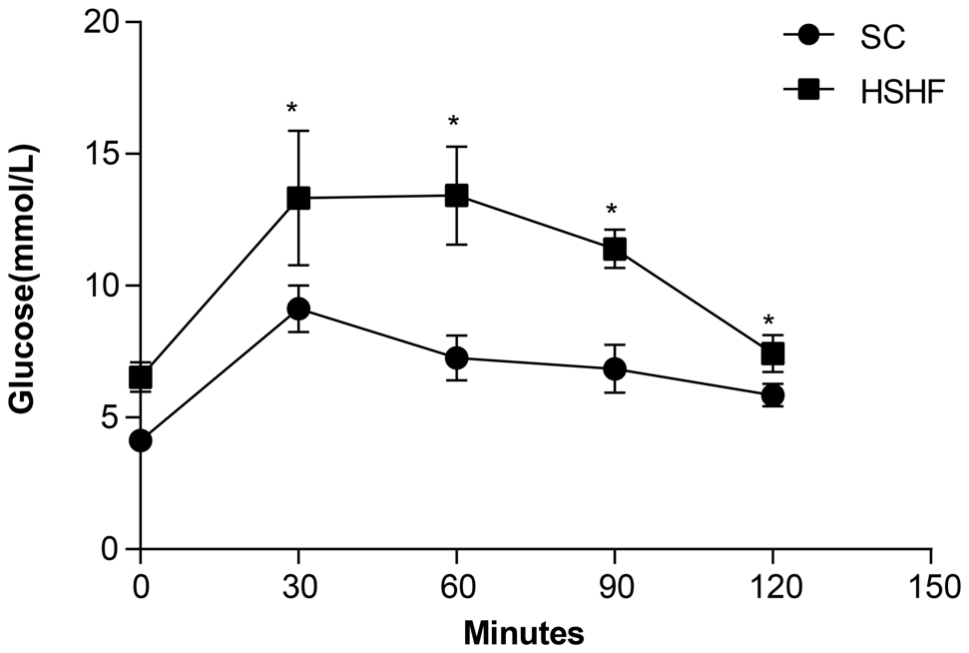
Intraperitoneal glucose tolerance test curve at 12 weeks. * p<0.05 vs SC group.

In the IPGTT, after 30 min of glucose administration, the plasma glucose of both groups increased to a maximum, although this maximum was higher is HSHF rats (p = 0.008). Glucose clearance in HSHF rats was delayed in comparison to SC rats, remaining high for 120 min (p<0.001) after glucose administration, which reflects glucose intolerance (Fig. 2).

### Changes of LPS, TNFα, IL-6, and the ALP content of the intestine tissue homogenates

As shown in Table 2, serum LPS, TNFα, IL-6, and the ALP content of 1% the small intestine tissue homogenates increased significantly at the 12^th^ week in HSHF rats compared to SC rats, and this difference continued to 48 weeks (Table 2). This finding suggests that intestinal endotoxemia and chronic inflammation occurred by the 12^th^ week and continued for 48 weeks in HSHF rats.

**Table 2:** Changes of LPS, ALP, and inflammation factors (x±s. n = 8).

Indexes	Groups	12^th^ week	24^th^ week	36^th^ week	48^th^ week
LPS, EU/ml	SC	0.07±0.01	0.07±0.01	0.08±0.01	0.08±0.01
	HSHF	0.67±0.07^a^	0.73±0.09^a^	0.74±0.07^a^	0.74±0.08 a
ALP, U/gprot	SC	0.015±0.008	0.019±0.007	0.02±0.013	0.019±0.016
	HSHF	0.05±0.017^a^	0.073±0.02^a^	0.075±0.02^a^	0.075±0.024^a^
TNFα, ng/ml	SC	1.09±0.21	1.02±0.13	7.24±1.48	6.56±3.5
	HSHF	2.01±0.75^a^	2.1±0.31^a^	1.96±0.31 a	1.93±0.30^a^
TNFα in pancreas, ng/ml	SC	0.02±0.01	0.02±0.004	0.019±0.01	0.018±0.005
	HSHF	0.02±0.01	0.03±0.02	0.15±0.04 ^abc^	0.17±0.045 ^abc^
TNFα in fat, ng/ml	SC	0.022±0.01	0.017±0.004	0.019±0.003	0.016±0.004
	HSHF	0.063±0.01 a	0.094±0.01 ^ab^	0.166±0.027 ^abc^	0.18±0.031 ^abc^
IL-6, pg/ml	SC	0.11±0.05	0.10±0.21	0.10±0.014	0.11±0.013
	HSHF	0.18±0.03^a^	0.24±0.03^a^	0.24±0.041^a^	0.25±0.042^a^

a p<0.05 vs normal control; ^b^ p<0.05 vs 12^th^ week group; ^c^ p<0.05 vs 24^th^ week group; ^d^ p<0.05 vs 36^th^ week group.

### Structure of intestinal tissue, liver, pancreas, and adipose tissue

**Figure 3:**
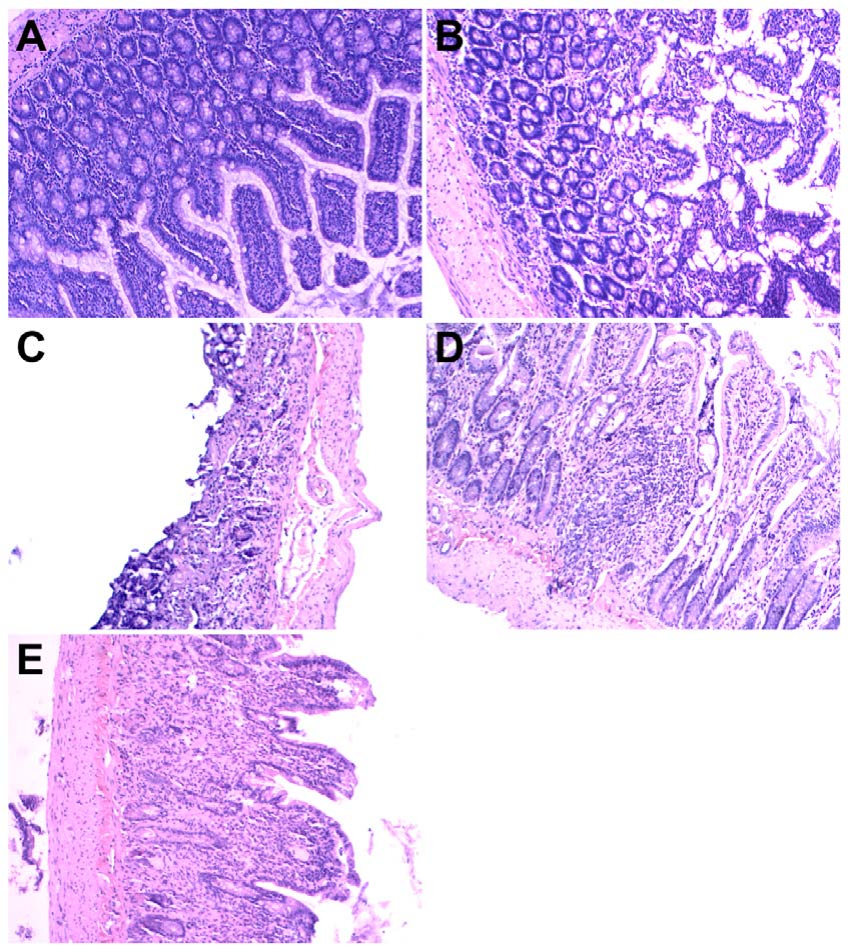
HE staining for intestine lesion. A. Normal morphology of intestine in standard chow group. B. Intestine lesion at 12 weeks in HSHF group. C. Intestine lesion at 24 weeks in HSHF group. D. Intestine lesion at 36 weeks in HSHF group. E. Intestine lesion at 48 weeks in HSHF group (×100).

SC rats had a normal intestinal structure. In contrast, as time progressed the specimens from the HSHF groups showed varying degrees of damage to the villus of the intestinal epithelium, including villus epithelial swelling, degeneration of the villous epithelium, collapse of the villi, and chronic inflammatory cell infiltration (Fig. 3A-E).

**Figure 4:**
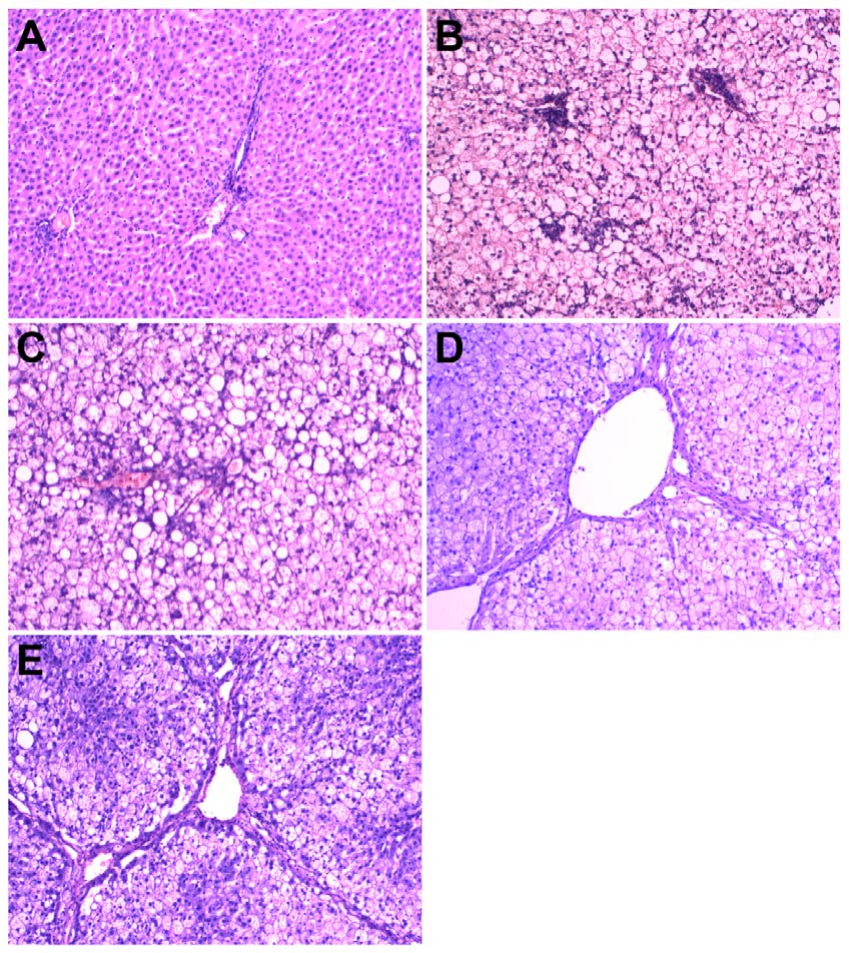
HE staining for histological examination of liver.

**Figure 5:**
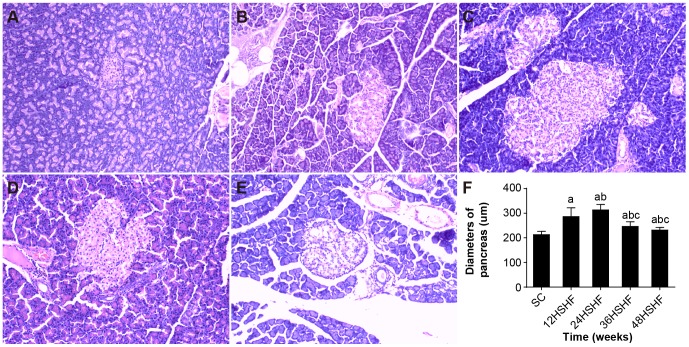
HE staining for histological examination of pancreas and comparison of pancreas diameters. A. Pancreas in SC group. B. Pancreas in 12 weeks in HSHF group. C. Pancreas in 24 weeks in HSHF group. D. Pancreas in 36 weeks in HSHF group. E. Pancreas in 48 weeks in HSHF group (HE staining, ×100). F. comparison of diameters of islet. ^a^ p<0.05 vs normal control; ^b^ p<0.05 vs 12^th^ week group; ^c^ p<0.05 vs 24^th^ week group; ^d^ p<0.05 vs 36^th^ week group.

SC rats had a normal liver structure (Fig. 4A). In HSHF rats, the liver was heavier (+97%, p = 0.001; +115%, p = 0.001; +120%, p = 0.002; +97.7%, p = 0.002) and showed gradually increased fatty degeneration, ballooning degeneration, and lobular and periportal inflammatory cell infiltration, starting at 12 weeks (Fig. 4B) and persisting throughout the study. Fibrosis gradually increased starting at the 24^th^ week (Fig. 4C, HYP content in liver compared with SC rats +75%, p = 0.03), and pseudolobules become apparent by the 36^th^ week (Fig. 4D, HYP content in liver compared with SC rats +120%, p = 0.004). Fibrosis showed further aggregation and presented as a woven network, so that normal liver tissue remodeling and liver cirrhosis occurred at 48 weeks (Fig. 4E, HYP content in liver compared with SC rats +150%, p = 0.004). Small or moderate lipid droplet depositions in the liver were clearly observed by the 12^th^ week in the HSHF rats (Sudan IV staining, Fig. 5A). Moderate and large lipid droplets were observed by the 24^th^ week in HSHF group and persisted until the 48^th^ week (Fig. 5B-D). As shown in Table 3, the FFAs in the liver were elevated at 12 weeks and peaked at 24 weeks.

**Table 3:** FFA in plasma, liver, pancreas and HYP in liver.

Indexs	Groups	12^th^ week	24^th^ week	36^th^ week	48^th^ week
FFA, mmol/L	SC	413.77±58.31	400.23±14.74	395.89±22.36	403.56±11.49
	HSHF	899.48±130.1^a^	1266.33±243.61^ab^	1343.07±345.66^ab^	1445.31±305.15 ^ab^
FFA in liver, mmol/L	SC	78.15±5.08	79.81±7.21	82.81±3.41	81.88±5.30
	HSHF	219.64±40.08^a^	268.01±51.13^ab^	296.31±30.44^ab^	302.53±22.48^ab^
FFA in pancreas, mmol/L	SC	41.48±1.85	40.89±5.53	48.81±5.36	44.98±6.93
	HSHF	43.05±5.04	84.20±3.19^ab^	103.2±11.46^abc^	134.77±11.71^abcd^
HYP in liver, ug/mg	SC	0.21±0.05	0.20±0.04	0.20±0.03	0.19±0.08
	HSHF	0.23±0.03	0.35±0.149^ab^	0.44±0.099^ab^	0.50±0.099^abc^

a p<0.05 vs normal control; ^b^ p<0.05 vs 12^th^ week group; ^c^ p<0.05 vs 24^th^ week group; ^d^ p<0.05 vs 36^th^ week group.

**Figure 6:**
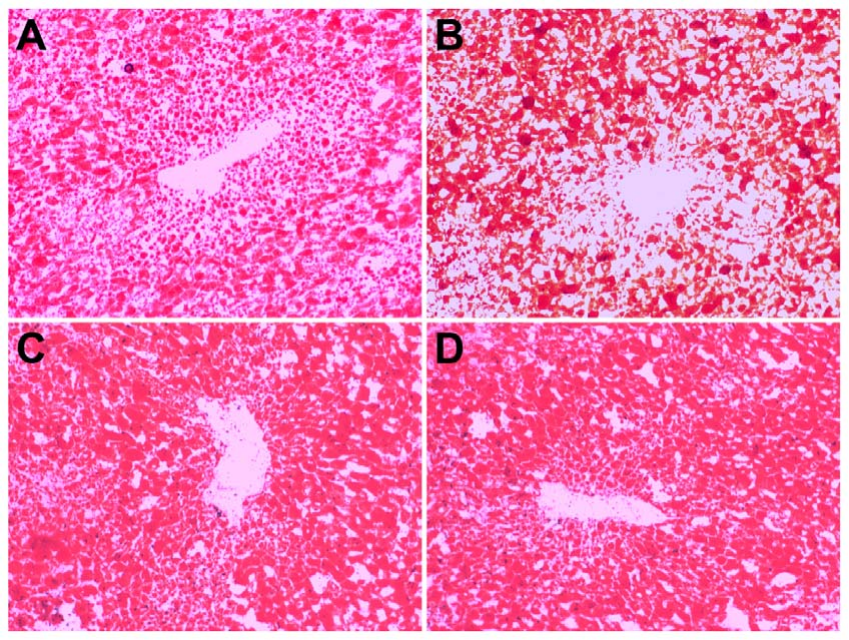
Sudan staining for liver. A. Hepatology in 12 weeks in HSHF group. B. Hepatology in 24 weeks in HSHF group C. Hepatology in 36 weeks in HSHF group. D. Hepatology in 48 weeks in HSHF group (Sudan staining, ×100).

**Figure 7:**
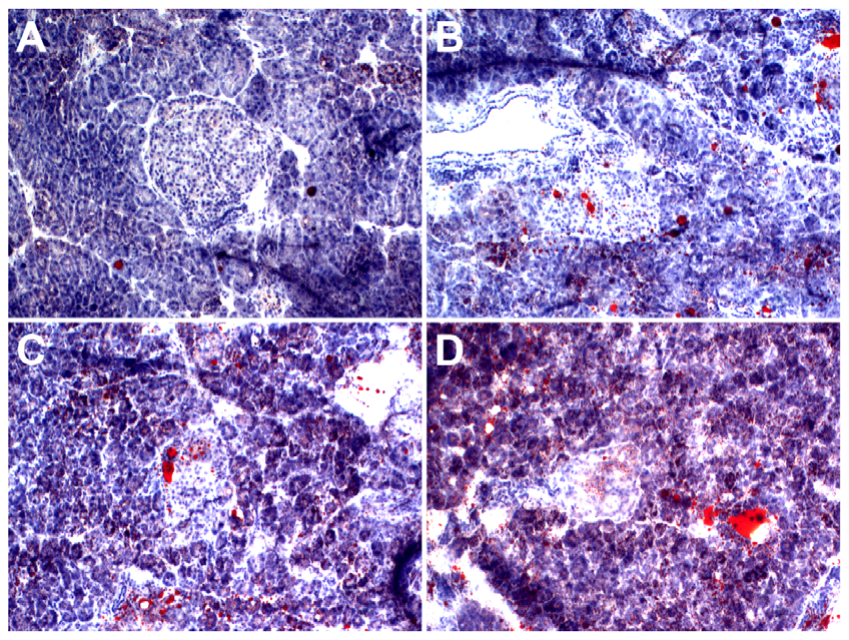
Sudan staining for pancreas. A. pancreas in 12 weeks in HSHF group; B. pancreas in 24 weeks in HSHF group; C. pancreas in 36 weeks in HSHF group; D. pancreas in 48 weeks in HSHF group (Sudan staining, ×100).

SC rats had a normal pancreas structure (Fig. 6A). The HSHF rats at the 12^th^ week displayed slightly increased pancreatic islets (Fig. 6B, islet diameter varied from 212.96±13.28 in SC rats to 286.30±35.40 in HSHF rats, +25.6%, p<0.001) and vascular engorgement. Islet diameter further increased (Fig. 6C, islet diameter varied from 210.40±14.27 in SC rats to 313.33±22.22 in HSHF rats, +32.85%, p<0.001) at the 24^th^ week, showing clear islet dilation. From weeks 36 to 48, the HSHF rats showed severe inflammatory cell infiltration surrounding these islets and islet fat deposition (Fig. 6D-E). The HYP content in the pancreas was elevated at the 36^th^ week (0.23±0.03 µg/mg, +21.1.%, p = 0.017) and peaked at the 48^th^ week (0.35±0.04 µg/mg, +94.4.%, p = 0.004). Half of the islets were significantly reduced at the 36^th^ week compared to the 24^th^ week in HSHF rats (islet diameter was 246.30±18.76 at the 36^th^ week, -27.21%, p<0.001) and further reduced at the 48^th^ week (232.55±10.51 at 48^th^ week, -34.73.%, p<0.001). Lipid droplets deposited in the pancreas were observed at 36 weeks, and lipid droplets deposited in the pancreas showed further aggregation over time (Fig. 7A-D). However, the FFAs in the pancreas were elevated at 24 weeks, progressively increasing up to 48 weeks (Table 3).

**Figure 8:**
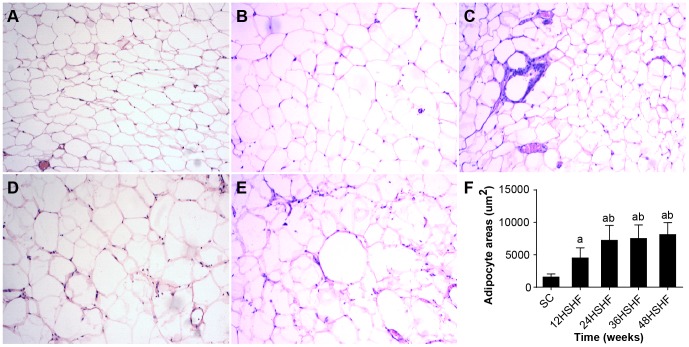
HE staining for histological examination of fat. A. fat in SC group. B. fat in 12 weeks in HSHF group. C. fat in 24 weeks in HSHF group. D. fat in 36 weeks in HSHF group. E. fat in 48 weeks in HSHF group (HE staining, ×100). F. comparison of adipocyte areas. ^a^ p<0.05 vs normal control; ^b^ p<0.05 vs 12^th^ week group; ^c^ p<0.05 vs 24^th^ week group; ^d^ p<0.05 vs 36^th^ week group.

The morphometry of adipocytes in HSHF rats displayed hypertrophy in comparison to SC rats from the 12^th^ week to the 48^th^ week. As early as the 24^th^ week, visible inflammatory cell infiltration was noted in the adipose tissue (Fig. 8A-E).

### TUNEL of liver and pancreas

**Figure 9:**
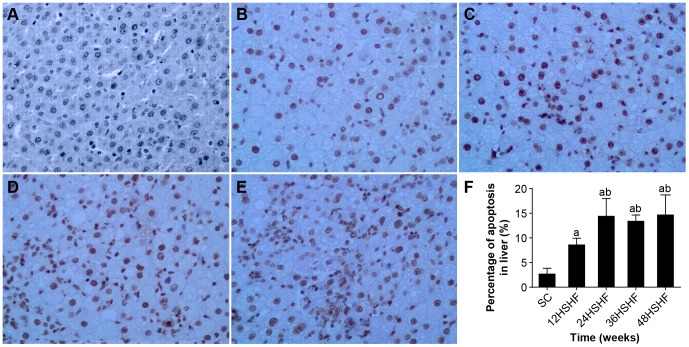
Observation of hepatocyte apoptosis. A. Hepatic cell in SC group. B. Hepatic cell in 12 weeks in HSHF group. C. Hepatic cell in 24 weeks in HSHF group. D. Hepatic cell in 36 weeks in HSHF group. E. Hepatic cell in 48 weeks in HSHF group (HE staining, ×100). F. Percentage of apoptosis in liver(%)^a^ p<0.05 vs normal control; ^b^ p<0.05 vs 12^th^ week group; ^c^ p<0.05 vs 24^th^ week group; ^d^ p<0.05 vs 36^th^ week group.

**Figure 10:**
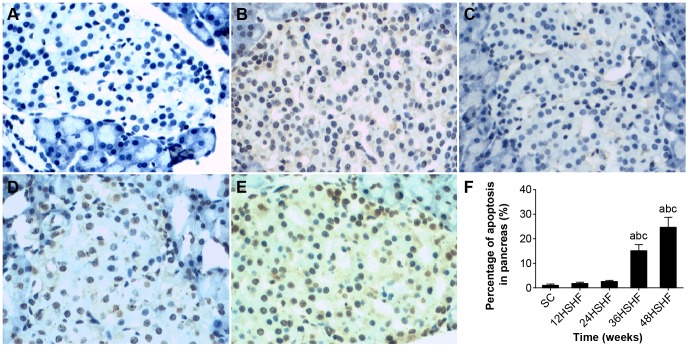
Observation of pancreas apoptosis. A. Pancreas in SC group. B. Pancreas in 12 weeks in HSHF group. C. Pancreas in 24 weeks in HSHF group. D. Pancreas in 36 weeks in HSHF group. E. Pancreas in 48 weeks in HSHF group (HE staining, ×100). F. Percentage of apoptosis in pancreas(%).^a^ p<0.05 vs normal control; ^b^ p<0.05 vs 12^th^ week group; ^c^ p<0.05 vs 24^th^ week group; ^d^ p<0.05 vs 36^th^ week group.

TUNEL showed that the SC group exhibits minimal hepatocyte apoptosis (Fig. 9A). However, hepatocyte apoptosis was significantly increased at 12 weeks in the HSHF group (Fig. 9B), peaked at 24 weeks in HSHF group (Fig. 9C), and continued to 48 weeks (Fig. 9D-E). TUNEL showed that islet apoptosis was significantly increased from 12 to 48 weeks in the HSHF group (Fig. 10A-E).

### CD68 and TNFαin adipose and pancreas

**Figure 11:**
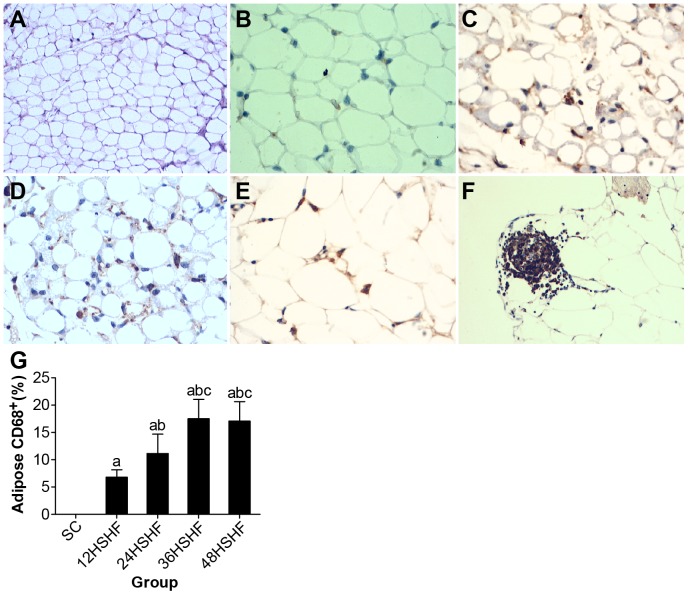
CD68 staining for adipose tissue. A. Normal morphology of fat in standard chow group (×200). B. 12 weeks in HSHF group (×400). C. 24 weeks in HSHF group (×400). D. 36 weeks in HSHF group (×400). E. 48 weeks in HSHF group (×400). F. 48 weeks in perivascular in HSHF group (×200). G. Adipose macrophage infiltration of rats in each group. ^a^ p<0.05 vs normal control; ^b^ p<0.05 vs 12^th^ week group; ^c^ p<0.05 vs 24^th^ week group; ^d^ p<0.05 vs 36^th^ week group.

Normal adipose tissue showed a very small number of infiltrating CD68-positive macrophages (Fig. 11A). However, adipose tissue inflammation and infiltration began to rise by the 12^th^ week (Fig. 11B) and gradually increased to the 48^th^ week in the HSHF group (Fig. 11C-E). Meanwhile, a large number of macrophages were present in the perivascular adipose tissue (Fig. 11F). These changes are consistent with changes in the TNFα content of adipose tissue (Table 2).

**Figure 12:**
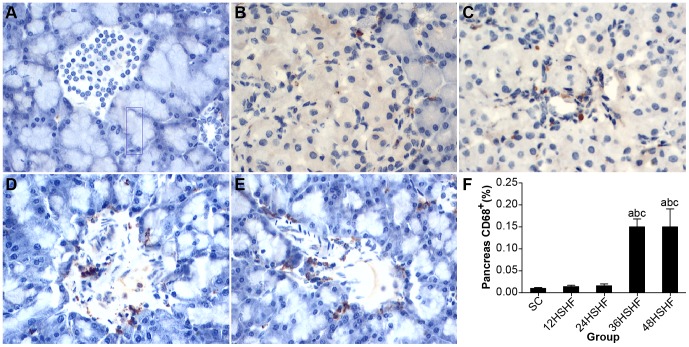
CD68 staining for pancreas. A. Normal morphology of fat in standard chow group (×100). B. 12 weeks in HSHF group. C. 24 weeks in HSHF group. D. 36 weeks in HSHF group. E. 48 weeks in HSHF group (×400). F. Pancreas macrophage infiltration of rats in each group. ^a^ p<0.05 vs normal control; ^b^ p<0.05 vs 12^th^ week group; ^c^ p<0.05 vs 24^th^ week group; ^d^ p<0.05 vs 36^th^ week group.

Marcophage infiltration was in a normal range for pancreas tissue collected from HSHF rats at 12 and 24 weeks (Fig. 12A-C). However, by 36 weeks macrophage infiltration was apparent in HSHF rats (Fig. 12D), which continued to the 48^th^ week in the HSHF group (Fig. 12E). As macrophage infiltration increased, so did the TNFα content of pancreas tissues (Table 2).

### Western blot results

**Figure 13:**
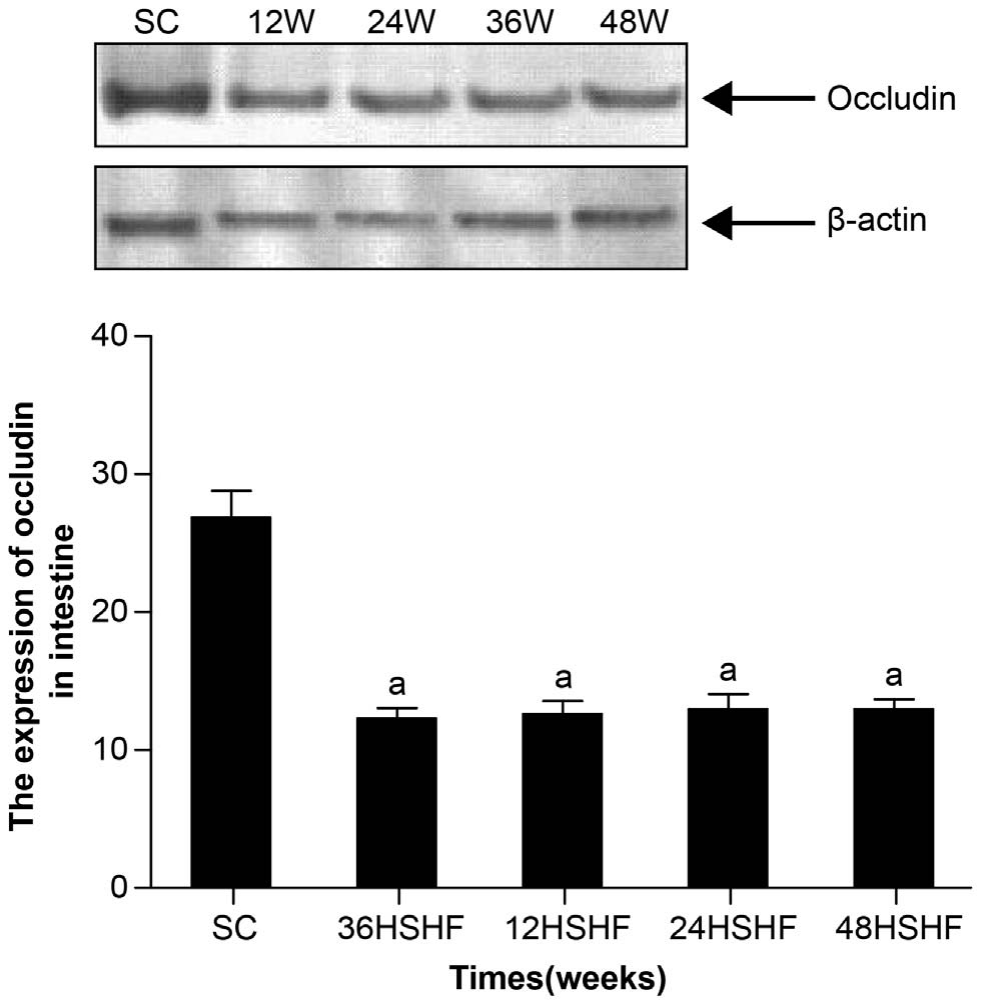
The protein expression of occludin in the intestine of rats. Standard control group (SC), 12th week group (12w), 24th week group (24w), 36th week group (36w), 48th week group (48w). ^a^ p<0.05 vs standard control group.

We found that a high fat and high sucrose diet led to about a 2-fold reduction in the expression occludin in the intestinal tissue by 12 weeks, a trend that continued to 48 weeks in HSHF group (p<0.001; Fig. 13).

## Discussion

Metabolic syndrome refers to central obesity, insulin resistance, impaired glucose tolerance, dyslipidaemia, and elevated blood pressure [18], [19]; these are considered to increase the incidence of cardiovascular disease and type II diabetes [20]-[22]. Metabolic syndrome is also closely related with an increased risk of NAFLD and kidney dysfunction [23], [24]. Metabolic syndrome and its related diseases present with chronic mild inflammation [25], [26]. The basic pathogenesis of metabolic syndrome is insulin resistance (IR) [27], [28]. Metabolic syndrome is widespread, occurring worldwide, and in recent years it has attracted much attention. The widespread occurrence of metabolic syndrome necessitates its urgent study to identify relevant causes and the signs of its occurrence and progression. These studies require viable animal models that adequately mimic all the aspects of the human disease, developing the key hallmarks of metabolic syndrome, especially obesity, diabetes, dyslipidaemia, hypertension, and possibly fatty liver disease [29]. Currently, transgenic mice or diet-induced mice are used as experimental animal models for the research of metabolic syndrome and its related diseases. Genetic models include db/db mice, ob/ob mice, Zucker diabetic fatty rats, and Otsuka Long-Evans Tokushima fatty rats. These models have been useful in assessing specific molecular mechanisms that may be involved in the development of obesity and related diseases, yet metabolic syndrome in humans is not a monogenetic disorder [29]. Therefore, it is important to determine whether these transgenic mice mimic human disease and whether these models display the features necessary to reflectively study human metabolic disease. For example, many of these models are based on mutations in the leptin gene and/or its receptor gene sequence. However, in humans, similar mutations represent a very rare recessive genetic disease, and until 2009, only four cases had been reported in a total of 15 people [30]. Animal models of diet-induced metabolic syndrome have classically focused on prepared metabolic syndrome induced by a pure fat diet [31]–[36] or pure high fructose diet [37]–[40]. However, the diet of the human is much more complicated. In modern daily life, people also eat foods containing high concentrations of sucrose, such as soft drinks and fast food products, and meat is widely consumed, which is often high in saturated fatty acids. Therefore, we have used a high sugar and high fat feeding regimen to monitor the health of rats over one year of diet. Rats on this diet develop obesity, dyslipidemia, impaired glucose tolerance, hyperglycemia, endotoxemia, NAFLD, and T2DM, as do humans. Using this animal model, we may systematically observe the occurrence and the development of metabolic disease and the relative role of endotoxin. We contend that metabolic syndromes arise from a common thread (diet), which gives important clues for the treatment of metabolic diseases in humans.

It is well known that obesity is an important pathogenic factor for metabolic diseases [41], [42]. Animal obesity may be induced by high glucose and high fat [43]–[45]. Obese fat cells get larger and hypertrophied adipocytes make a number of lipids, such as the TG, DAG, and FFA that may accumulate in non-fatty tissues, such as the liver and pancreas [46]. According to our experimental observations, TAG and FFA begin to accumulate in the liver by 12 weeks, causing severe lipid degeneration and fatty liver hepatitis. By 36 weeks, the liver becomes completely saturated with lipids, and then both fibrosis and cirrhosis may arise. At 36 weeks, these lipids (TG, FFA) flow into the pancreas following circulation and are deposited in islet β-cells in the pancreas and may negatively alter the biosynthesis, secretion and apoptosis of β-cells. At the same time, HOMA-β is significantly decreased in HSHF rats, promoting the development of T2DM [47]. Hypertrophied fat cells also may secret abnormal levels of pro-inflammatory cytokines. This can result in the excessive production/release of TNF-α and C-reactive protein (CRP). We found that TNF-α in the adipose tissue of HSHF rats begins to increase by the 12^th^ week and reaches a peak by the 36^th^ week, which may be associated with insulin resistance (IR). At the same time, the release of insulin-sensitive cytokines, such as adiponectin, is often dampened, exacerbating IR [48]. In addition, hypertrophied fat cells can cause ischemia and necrosis due to an apparent decrease in hemoperfusion [49]. Necrotic adipocytes, by the action of MCP-1, can induce monocytes to migrate out from the intravascular tissue and infiltrate the damaged areas of adipose tissue where they transform into macrophages [49]. When monocytes differentiate into macrophages, more pro-inflammatory mediators may be released, further aggravating IR. We have also seen in this in the course our experiment. CD68 is expressed in HSHF adipose tissue at 12 weeks and gradually increases over the 48 week timeframe, while TNFα also increases.

NAFLD and T2DM are closely related to metabolic syndrome. The chief characteristics of these diseases are a persistent low-grade inflammatory state and insulin resistance. Recent studies have shown that patients with NAFLD and T2DM often have increased LPS levels in their plasma [5], [7], [8]. However, how LPS influences metabolic diseases warrants further study. Our work shows that high levels of LPS may be maintained in the plasma of rats fed a high sugar and high fat diet. While chronic low-grade inflammation occurred throughout the study in these rats, the levels of glucose and insulin in the blood increased by 12 weeks of modeling. At the same time, the insulin resistance index increased significantly and was maintained at a high level. Concomitantly, the secretion index of β-cells was progressively reduced. These findings suggest that the HSHF rats were in a chronic state of low-grade inflammation accompanied by metabolic changes (as early as 12 weeks), denoting an early occurrence of diabetes. Late pathological observations showed islet atrophy and an overall reduction of in the number of islet cells, highlighting a progressive decline of secretive function, until secretory failure, which resulted in T2DM.

Although our model may be time-consuming, it may more accurately reflect the conditions experienced in humans and could be readily used to model metabolic syndrome, NAFLD, and T2DM, simply by placing the rats on a high sugar and high fat diet, as would be consumed in the western world. This model can now be implemented to further study MS, its related diseases, and how they arise.
